# Identification of memory B-cell-associated miRNA signature to establish a prognostic model in gastric adenocarcinoma

**DOI:** 10.1186/s12967-023-04366-2

**Published:** 2023-09-21

**Authors:** Ruquan Liu, Biaojie Huang, Yongzhao Shao, Yongming Cai, Xi Liu, Zhonglu Ren

**Affiliations:** 1 School of Medical Information and Engineering, Guangdong Pharmaceutical University, Guangzhou, 510006 China; 2 Guangdong Province Precise Medicine Big Data of Traditional Chinese Medicine Engineering Technology Research Center, Guangzhou, 51006 China; 3 Department of Population Health, New York University Grossman School of Medicine, New York, NY 10016 USA

## Abstract

**Background:**

Memory B cells and microRNAs (miRNAs) play important roles in the progression of gastric adenocarcinoma (GAC), also known as stomach adenocarcinoma (STAD). However, few studies have investigated the use of memory B-cell-associated miRNAs in predicting the prognosis of STAD.

**Methods:**

We identified the marker genes of memory B cells by single-cell RNA sequencing (scRNA-seq) and identified the miRNAs associated with memory B cells by constructing an mRNA‒miRNA coexpression network. Then, univariate Cox, random survival forest (RSF), and stepwise multiple Cox regression (StepCox) algorithms were used to identify memory B-cell-associated miRNAs that were significantly related to overall survival (OS). A prognostic risk model was constructed and validated using these miRNAs, and patients were divided into a low-risk group and a high-risk group. In addition, the differences in clinicopathological features, tumour microenvironment, immune blocking therapy, and sensitivity to anticancer drugs in the two groups were analysed.

**Results:**

Four memory B-cell-associated miRNAs (hsa-mir-145, hsa-mir-125b-2, hsa-mir-100, hsa-mir-221) with significant correlations to OS were identified and used to construct a prognostic model. Time-dependent receiver operating characteristic (ROC) curve analysis confirmed the feasibility of the model. Kaplan‒Meier (K‒M) survival curve analysis showed that the prognosis was poor in the high-risk group. Comprehensive analysis showed that patients in the high-risk group had higher immune scores, matrix scores, and immune cell infiltration and a poor immune response. In terms of drug screening, we predicted eight drugs with higher sensitivity in the high-risk group, of which CGP-60474 was associated with the greatest sensitivity.

**Conclusions:**

In summary, we identified memory B-cell-associated miRNA prognostic features and constructed a novel risk model for STAD based on scRNA-seq data and bulk RNA-seq data. Among patients in the high-risk group, STAD showed the highest sensitivity to CGP-60474. This study provides prognostic insights into individualized and precise treatment for STAD patients.

**Supplementary Information:**

The online version contains supplementary material available at 10.1186/s12967-023-04366-2.

## Background

Gastric cancer (GC) is the fifth most common cancer in the world and the fourth leading cause of cancer-related death [1]. Gastric adenocarcinoma (GAC), also known as stomach adenocarcinoma (STAD), is the most common histological type of GC [2]. Risk factors for GC include Helicobacter pylori infection, Epstein‒Barr virus infection, dietary factors, tobacco use, obesity, and radiation exposure [3]. In addition, gene mutations, chromosomal alterations, transcriptional disorders, and epigenetic modifications are important common factors of carcinogenesis [4]. Although some progress has been made in the treatment of STAD in recent years, the therapeutic effect against advanced disease is poor and unsatisfactory [5–7]. In the treatment of advanced disease, only trastuzumab and some immune checkpoint inhibitors, such as nivolumab and pembrolizumab, have shown consistent and reliable efficacy in patients with HER2-positive and PDL1-positive tumours based on chemotherapy [8]. Currently, individualized cancer therapy has become a new focus of cancer treatment, and more in-depth research on STAD and the development of new effective molecular signatures for predicting the immunotherapy response and prognosis of STAD patients are necessary.

Most studies of prognostic signatures for STAD have focused on protein-coding genes, whereas few studies have investigated genes for noncoding RNA. Increasing evidence has confirmed that miRNAs directly or indirectly regulate the expression of target genes and play an important role in the pathological development of STAD [9]. According to previous studies, miRNAs can promote the proliferation of tumour cells; for example, miR-21 can enhance the proliferation and invasion of gastric cancer cells by targeting the expression of *PTEN* [10]. MiR-148a and miR-196a inhibit the expression of *p27* and promote the proliferation of gastric cancer cells [11, 12]. Hypoxia-induced miR-224 promotes the growth of gastric cancer cells by downregulating *RASSF8* [13]. Moreover, miRNAs can inhibit the activity and proliferation of tumour cells; for example, miR-375 inhibits the activity of gastric cancer cells by downregulating *PDK1* or 14-3-3 ζ [14]. MiR-135a may inhibit the activation of *p-STAT3* by targeting *JAK*, reduce the expression of *Cyclin D1* and *BCL-XL*, and inhibit the proliferation of gastric cancer cells, thus playing the role of tumour inhibitor [15]. MiR-15a and miR-16-1 downregulate *YAP1* and inhibit the proliferation and migration of gastric cancer cells [16]. Therefore, mining miRNAs for a new type of STAD prognostic marker is a promising research direction.

Previous studies have shown that B cells play an important role in tumour progression; for example, compared with that in peripheral blood, the number of memory B cells and antibody-secreting B cells in the tumour microenvironment (TME) is increased, and the expression of B-cell surface markers is also different, e.g., *CD23* is downregulate and *CD86* is upregulated in breast cancer [17]. An increase in the frequency of B cells secreting IL-35 has been reported in patients with advanced gastric cancer [18]. Moreover, many studies have shown that B cells play an important role in immune checkpoint inhibitor treatment of cancer [19–22]. However, few studies have focused on the use of memory B-cell-associated miRNAs to predict the prognosis of patients with STAD.

**Fig. 1:**
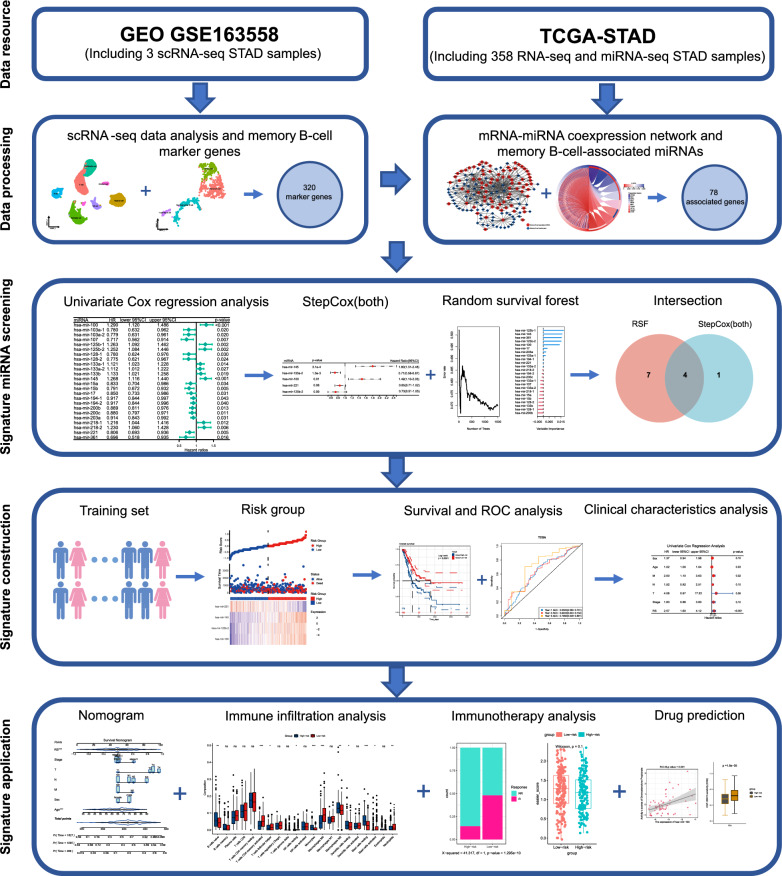
Flowchart of this study

In recent years, the development of scRNA-seq technology has enabled researchers to explore the heterogeneity of tumours at the cellular level [23], which provides a new method for studying tumour biomarkers. Previous studies have focused on identifying bulk RNA-seq biomarkers by mining STAD data [24–26]. In this study, we integrated scRNA-seq and bulk RNA-seq to establish a prognostic signature of miRNAs associated with memory B cells, which predicted the prognosis and immunotherapy effect in STAD patients, revealed the difference in the TME based on risk grouping, and predicted potentially new effective anticancer drugs. The flow chart of this study is shown in Fig. 1.

## Methods

### Data collection

We downloaded the single-cell transcriptome profile (GSE163558) of STAD from the Gene Expression Omnibus (GEO) database (www.ncbi.nlm.nih.gov/) [27], and three primary gastric cancer samples were selected to identify the marker genes of memory B cells. The mRNA expression data, miRNA expression data and corresponding clinical STAD data were downloaded from The Cancer Genome Atlas (TCGA) database (www.cancer.gov/) [28]. A total of 358 tumour samples with mRNA and miRNA expression data and complete survival information were obtained.

### Identification of memory B-cell-associated miRNAs in STAD patients

We used the "Seurat" R package to analyse the scRNA-seq data [29], control the quality of the data, remove genes with less than 3 cell counts, cells with less than 200 genes and cells with more than 5% mitochondrial genes, and then normalize them. Principal component analysis (PCA) was used to extract the first 15 principal components, and uniform manifold approximation and projection (UMAP) was used for cell clustering. Referring to the uploader's cluster notes [27], the "FindAllMarkers" function was used to find the marker genes of memory B cells, and the critical threshold of P < 0.05 was set. The related genes of memory B cells were obtained. The correlation between TCGA-STAD mRNA expression data and miRNA expression data was assessed using Pearson’s correlation coefficient, and the correlation between memory B-cell-associated genes and miRNA was calculated. We determined that miRNAs with an absolute correlation coefficient greater than 0.5 and P < 0.01 were memory B-cell-associated miRNAs [30]. Cytoscape software was used to visualize the memory B-cell-associated mRNA‒miRNA coexpression network [31]. We also used miEAA 2.0 (https://ccb-compute2.cs.uni-saarland.de/mieaa2/) to analyse the enrichment of transcription factors in memory B-cell-associated miRNAs [32].

### Construction of an STAD prognostic signature using memory B-cell-associated miRNAs

Univariate Cox proportional hazard regression analysis was used to evaluate the prognostic value of memory B-cell-associated miRNAs in the assessment of overall survival (OS) in 358 TCGA-STAD patients. MiRNA was screened from memory B-cell-associated miRNAs with significant prognosis. Then, random survival forest (RSF) was used to rank the importance of memory B-cell-associated miRNAs with significant prognosis value, and miRNAs with an importance > 0 were selected for subsequent analysis. Memory B-cell-associated miRNAs with significant prognosis values were screened by StepCox(both) analyses. The miRNAs identified by both RSF and StepCox(both) as memory B-cell-associated miRNAs significantly associated with OS were included in multivariate Cox regression analysis, and the memory B-cell-associated miRNA risk score (RS) of each STAD patient was calculated based on the following formula:

$$ RS = \mathop \sum \limits_{i = 1}^{n} [coefficient\left( {miRNA} \right) \times expression\left( {miRNA} \right)] $$

According to the median RS, all patients were divided into a high-risk group and a low-risk group. Kaplan‒Meier (K‒M) survival curves and log-rank tests were used to analyse and compare survival between the low-risk group and the high-risk group. The 1-, 3- and 5-year ROC curves were drawn using the "survivalROC" R package to evaluate the prognostic value of the RS over time. In addition, 358 TCGA-STAD patients were randomly assigned to validation cohort 1 and validation cohort 2. Prognostic characteristics in the two validation cohorts were assessed by K‒M survival analysis and ROC curve analysis.

To further understand the clinical significance of the miRNA-based RS, univariate Cox proportional hazard regression analysis was used to compare the predictive accuracy of age, sex, stage, and RS on the prognosis of individuals with STAD. In addition, multivariate Cox proportional hazard regression analysis was used to determine whether age, sex, stage, and RS of STAD patients could be used as independent prognostic factors.

Based on the RS and clinical characteristics of the TCGA sample, we used the "survival" and "rms" R packages to construct a nomogram and evaluated the performance of the nomogram using calibration curves and ROC curves.

### Analysis of clinicopathological characteristics, TME and immunotherapy

We generated summary statistics for different clinical characteristics of TCGA-STAD patients based on the RS and analysed the survival of high- and low-risk groups based on different clinical characteristics. In terms of the TME, we used the "ESTIMATE" R package to evaluate the immune scores of different risk groups [33] and the "CIBERSORT" R package to calculate the differences in immune cell infiltration among different risk groups [34]. The efficacy of anti-PD-L1 treatment in patients with STAD was predicted based on the Tumour Immune Dysfunction and Exclusion (TIDE) scores evaluated as described at the website (http://tide.dfci.harvard.edu/), and the TIDE score for each sample in the high- and low-risk groups was calculated [35]. In addition, to predict the possibility of STAD responding to immune checkpoint blockade (ICB) therapy, the immunotherapy response score of each patient was calculated using the "EaSIeR" R software package [36]. This method integrates quantitative descriptive information about the tumour mutational burden (TMB) and immune microenvironment to predict the immune response. EaSIeR provides the possibility that each patient will benefit from ICB treatment; thus, the higher the relative score is, the more likely the patient’s cancer will respond to immunotherapy.

### Drug sensitivity analysis and screening

We aimed to further identify new and more effective drugs for the treatment of STAD by using the CellMiner database (https://discover.nci.nih.gov/cellminer/) to screen anticancer drugs with a significant correlation between sensitivity and prognostic miRNA [37]. We also used the "pRRophetic" R software package (https://osf.io/5xvsg/) to predict the semi-maximum inhibitory concentration (IC50) of different drugs in the high- and low-risk groups [38]. The lower the IC50 of a drug is, the more effective the drug will be in treating cancer.

### Statistical analysis

All statistical analyses were carried out using the R software version 4.2.1 (http://www.R-project.org). Cox regression and K‒M curves were used for survival analysis. The Wilcoxon test was used to compare the quantitative differences between the two groups (except when special instructions stated otherwise). P < 0.05 was considered to be statistically significant.

## Result

### Analysis of scRNA-seq data and identification of memory B cells in STAD patients

**Fig. 2:**
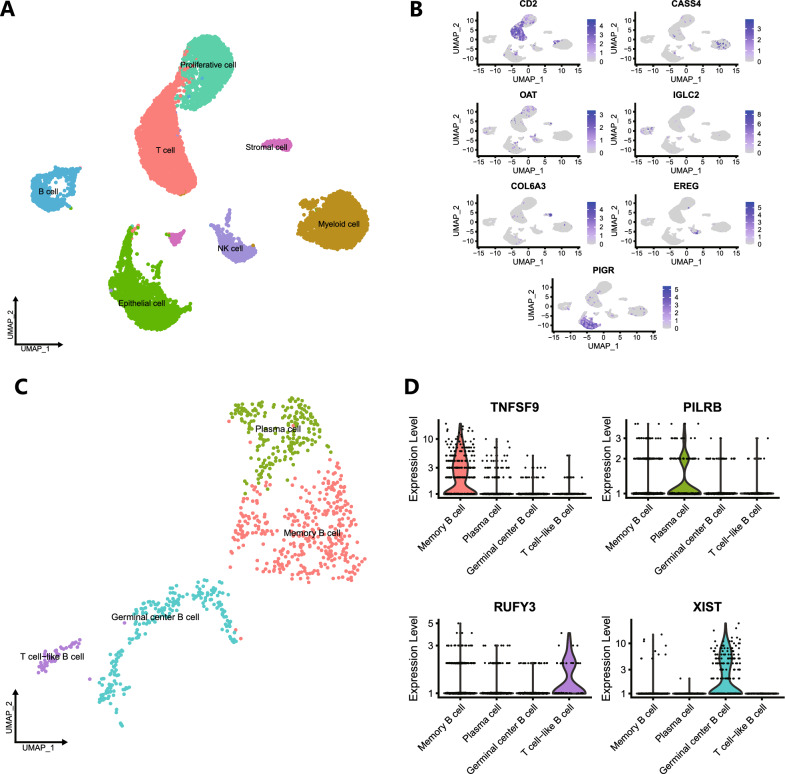
Identification of B-cell marker genes by scRNA-seq analysis. **A** After the first-level classification, seven cell types were identified by marker gene annotation. **B** UMAP plot showing the expression of the marker genes of the seven cell types. **C** After the second-level classification of B cells, four cell types were identified by marker gene annotation. **D** Violin plot showing the expression of the marker genes of the four cell types

We obtained the gene expression profiles of three primary gastric cancer samples from GSE163558. After quality control, normalization and dimensionality reduction by the PCA method, according to the known marker genes and the literature evidence of data uploaders, all cells were annotated as seven cell types: epithelial cells, proliferative cells, T cells, B cells, natural killer cells, and myeloid cells (Fig. 2A). The UMAP plot was used to show the expression level of marker genes in the seven known types of cells (Fig. 2B). Then, B cells were further subclustered into four subgroups: memory B cells, plasma cells, germinal center B cells and T-cell-like B cells (Fig. 2C). A violin plot was used to show the expression levels of marker genes of four known B-cell subsets (Fig. 2D). We identified 320 memory B-cell marker genes for subsequent analysis (Additional file 1: Table S1).

### Identification and enrichment analysis of memory B-cell-associated genes and miRNAs

**Fig. 3:**
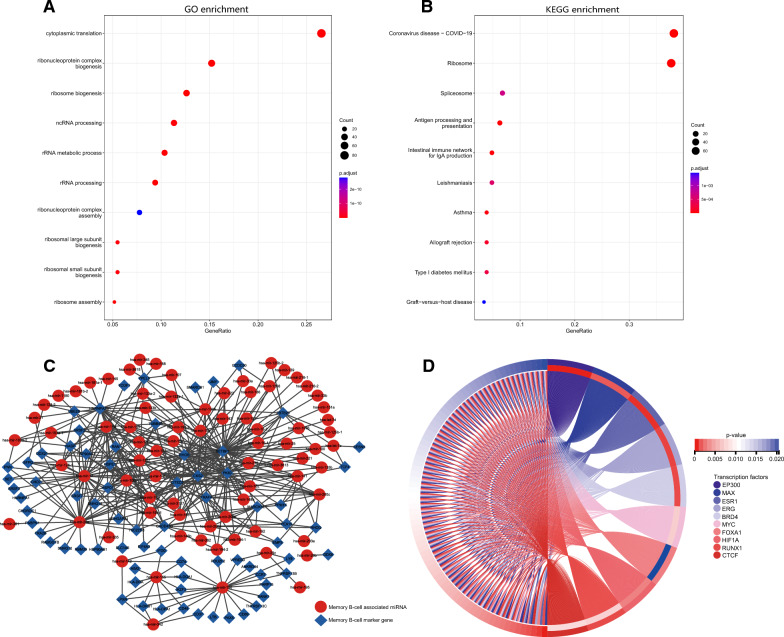
Enrichment analysis and the coexpression network of memory B-cell-associated genes. **A** GO enrichment analysis of memory B-cell-associated genes. **B** KEGG enrichment analysis of memory B-cell-associated genes. **C** Coexpression network of memory B-cell-associated genes and miRNAs. **D** Transcription factor enrichment analysis of memory B-cell-associated miRNAs

We screened the marker genes of memory B cells as memory B-cell-associated genes. Gene Ontology (GO) enrichment analysis of these memory B-cell-associated genes showed that they were enriched in cytoplasmic translation, ribosomal protein complex production, noncoding RNA treatment, rRNA metabolism and processing (Fig. 3A). Kyoto Encyclopedia of Genes and Genomes (KEGG) enrichment analysis showed that they were mainly enriched in ribosomes, splicing bodies, antigen processing and presentation, and IgA production in intestinal immune networks (Fig. 3B). Based on the miRNA expression profile and memory B-cell-associated gene expression profile of TCGA-STAD patients, Pearson’s correlation analysis was carried out, and a coexpression network was constructed (Fig. 3C). We identified 78 strongly associated memory B-cell-associated miRNAs (Additional file 2: Table S2). Enrichment analysis using the ORA algorithm showed that the 78 miRNAs were mainly enriched in transcription factors such as *EP300*, *MAX*, *ESR1*, *ERG*, *BRD4*, *MYC*, *FOXA1*, *HIF1A*, *RUNX1*, and *CTCF* (Fig. 3D).

### Screening of STAD memory B-cell-associated prognostic miRNAs

**Fig. 4:**
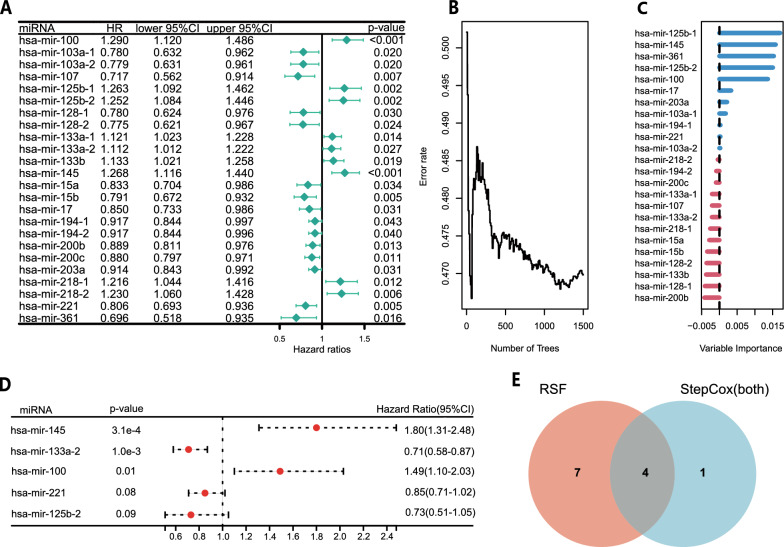
Screening of memory B-cell-associated prognostic miRNAs. **A** Screening candidate memory B-cell-associated miRNAs by univariate Cox regression analysis. **B**–**C** Random survival forest error rate versus the number of classification trees and the relative importance of 24 miRNAs. **D** Multivariate stepwise Cox regression analysis screened five prognosis-associated miRNAs from candidate miRNAs. **E** Venn diagram showed four miRNAs screened by both RSF and StepCox(both)

First, through univariate Cox regression analysis, we found that 24 STAD memory B-cell-associated miRNAs were significantly correlated with the prognosis of TCGA-STAD patients (Fig. 4A). Then, 11 miRNAs with relative importance > 0 were identified using RSF, namely, hsa-mir-125b-1, hsa-mir-145, hsa-mir-361, hsa-mir-125b-2, hsa-mir-100, hsa-mir-17, hsa-mir-203a, hsa-mir-103a-1, hsa-mir-194-1, hsa-mir-221 and hsa-103a-2 (Fig. 4B, C), and five miRNAs, namely, hsa-mir-145, hsa-mir-133a-2, hsa-mir-125b-2, hsa-mir-100 and hsa-mir-221, were identified by StepCox(both) analysis (Fig. 4D). Finally, four shared miRNAs (hsa-mir-145, hsa-mir-125b-2, hsa-mir-100 and hsa-mir-221) of RSF and StepCox(both) were identified as memory B-cell-associated prognostic miRNAs (Fig. 4E).

### Construction and validation of the prognostic signature of memory B-cell-associated miRNAs

**Fig. 5:**
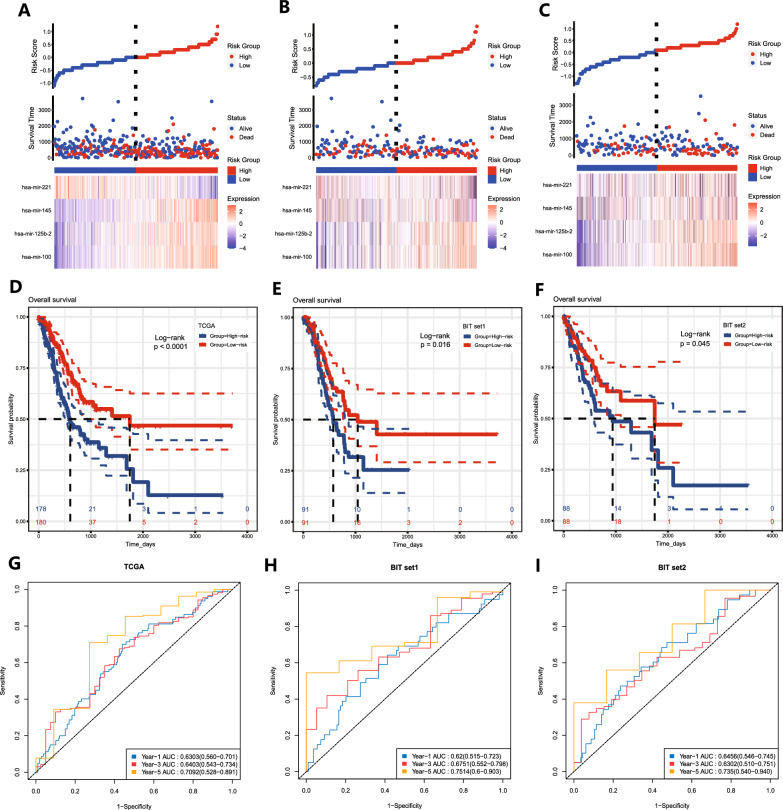
Construction and validation of the memory B-cell-associated miRNA prognostic signature. **A–C** RS distribution, survival status, and heatmap of the expression of four memory B-cell-associated miRNAs in the high-risk and low-risk groups of the training cohort, validation cohort 1, and validation cohort 2. **D–F** K‒M survival analysis of three cohorts. **G–I** Time-dependent ROC curve analysis of three cohorts

The Pearson’s correlation coefficients were determined using multivariate Cox regression analysis, and the associated RS was calculated from the Cox regression model as follows: RS= (0.29590272 * hsa-mir-100 expression) + (-0.19295689 * hsa-mir-125b-2 expression) + (0.17369659 * hsa-mir-145 expression) + (-0.07541793 * hsa-mir-221 expression). According to the median RS, all patients were divided into high-risk and low-risk groups. hsa-mir-221 was highly expressed in the high-risk group, and hsa-mir145, hsa-mir-100, and hsa-mir-125b-2 were highly expressed in the low-risk group (Fig. 5A). K‒M curve analysis showed that patients in the high-risk group had a poorer prognosis (P<0.0001) (Fig. 5D). ROC curve analysis was used to assess the predictive ability of the model, and the AUC values and 95% confidence intervals (CIs) for predicting OS at 1, 3, and 5 years were 0.6303 (0.560-0.701), 0.6403 (0.543-0.734), and 0.7092 (0.528-0.891), respectively (Fig. 5G). Subsequently, TCGA-STAD patients were randomly divided into two groups in a 1:1 ratio, and we used these two groups as a validation cohort for internal validation. Patients were divided into high-risk and low-risk groups according to the median RS (Fig. 5B, C), and the results showed that the high-risk group had a poorer prognosis in both validation cohorts (Fig. 5E, F). ROC curve analysis further confirmed the stability of the RS model, validation cohort 1, with AUCs and 95% CIs for predicting OS at 1, 3, and 5 years of 0.6200 (0.515–0.723), 0.6751 (0.552–0.798), and 0.7514 (0.600–0.903), respectively; for validation cohort 2, the AUCs and 95% CIs for predicting OS at 1, 3, and 5 years were 0.6456 (0.546–0.745), 0.6302 (0.510–0.751), and 0.7350 (0.540–0.940), respectively (Fig. 5H, I).

### RS-based clinical analysis and construction of the nomogram

**Fig. 6:**
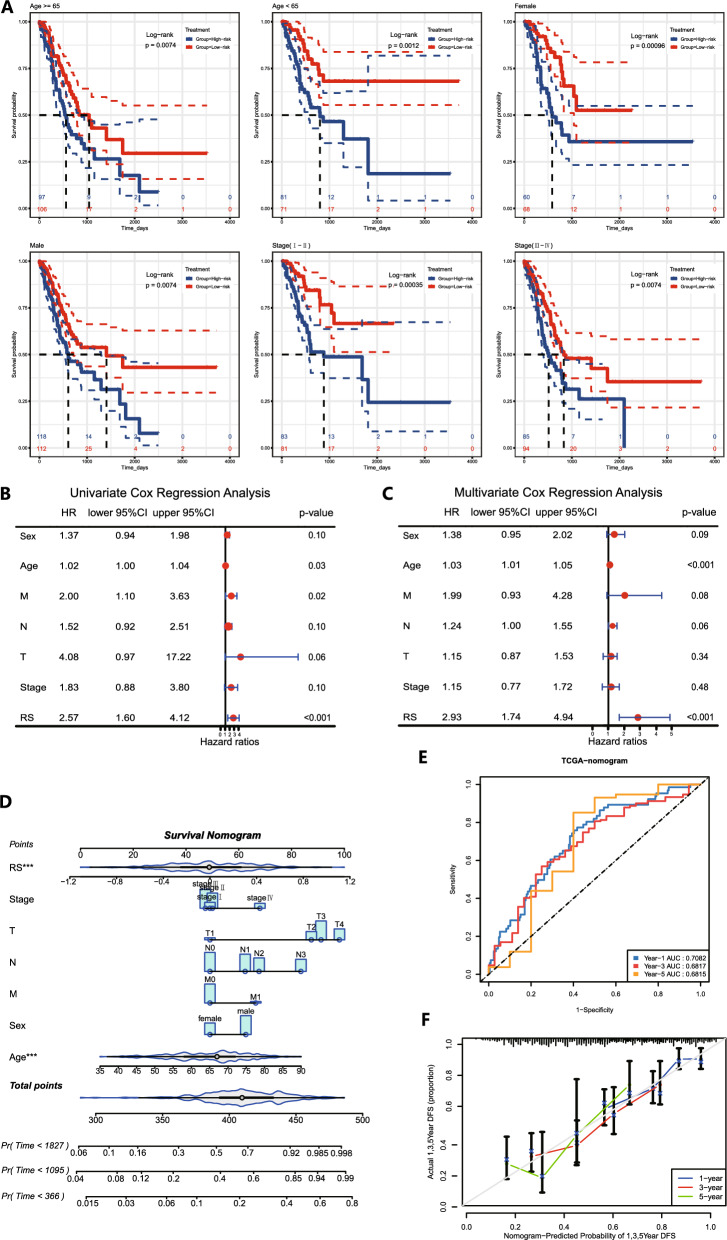
RS-based clinical analysis and construction of the nomogram. **A** K‒M survival analysis of survival stratified by age, sex and stage. **B–C** The results of univariate Cox regression analysis and multivariate Cox regression analysis of clinical characteristics and risk scores. **D** Nomogram based on RS and clinical characteristics. **E** ROC curve analysis results. **F** Calibration curve analysis results. *** P<0.001

We found that the high-risk and low-risk groups differed in both clinicopathological characteristics and OS. There were no significant differences between the high-risk and low-risk groups in terms of sex, age and distribution of tumour metastasis (M stage), while there were significant differences in terms of lymph node involvement (N stage), depth of tumour infiltration (T stage) and tumour grade (Stage), and the proportion of patients with N3 stage, T4 stage and Stage II disease was significantly higher in the high-risk group than in the low-risk group (Additional file 3: Table S3). We divided STAD patients into different subgroups according to the index of clinicopathological characteristics for survival analysis, and the K‒M curves showed that patients in the high-risk group had a worse prognosis than those in the low-risk group, whether grouped by sex, age, TNM stage or Stage (Fig. 6A). In addition, we explored the clinical application of the RS in patients with STAD using univariate Cox regression and multivariate Cox regression analyses. Univariate Cox and multivariate Cox results showed (Fig. 6B, C) that the RS was significantly associated with prognosis and was an independent factor affecting survival.

To improve the clinical utility of the model, a nomogram was constructed based on our risk scores for prognostic characteristics and other clinicopathological indicators of patients to provide a more comprehensive prediction of patient OS (Fig. 6D). The results of the ROC curve of the nomogram showed reliable performance with AUCs of 0.7082, 0.6817 and 0.6815 (Fig. 6E). The calibration curve was used to test the consistency of the predicted and actual risks of the prediction model, and the results showed that the predicted OS of the nomogram was close to the actual OS probability (Fig. 6F).

### Analysis of the tumour microenvironment based on the RS

**Fig. 7:**
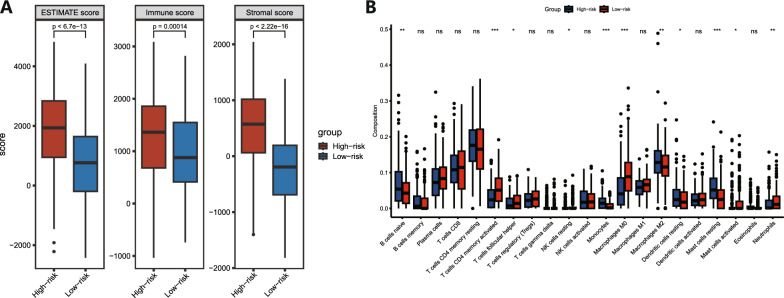
RS-based analysis of the tumour immune microenvironment. **A** Differences in the estimate, immune, and stromal scores between the low-risk and high-risk groups. **B** Differential expression levels of 22 types of tumour-infiltrating immune cells between the low-risk and high-risk groups. * P<0.05, ** P<0.01, *** P<0.001, ns not significant

To investigate the relationship between the RS and TME, we used the ESTIMATE algorithm to calculate the stromal score, immune score and ESTIMATE score of STAD patients, and the results showed that the high-risk group had significantly higher scores than the low-risk group (Fig. 7A). In terms of immune cells, the results of the CIBERSORT algorithm showed that the contents of naïve B cells, monocytes, M2 macrophages, resting dendritic cells and resting mast cells in the high-risk group were significantly higher than those in the low-risk group, whereas activated memory CD4 T cells, follicular helper T cells, resting NK cells, M0 macrophages, activated mast cells and neutrophils were significantly lower than those in the low-risk group (Fig. 7B).

### Analysis of immunotherapy based on the RS

**Fig. 8:**
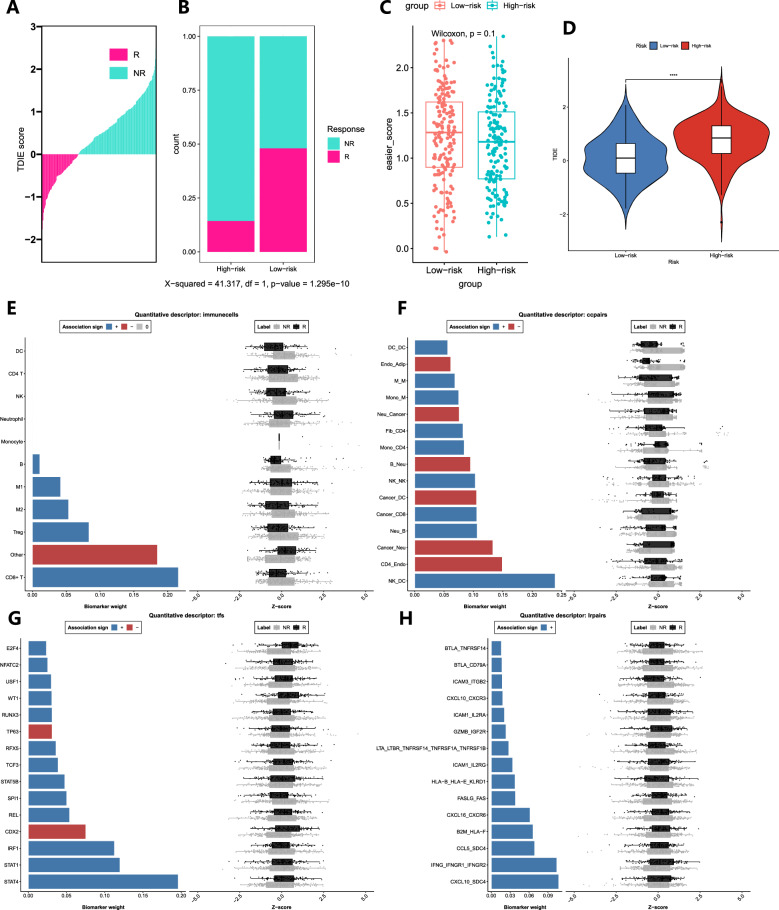
RS-based analysis of ICB immunotherapy response. **A** Result of ICB immunotherapy response prediction in TCGA-STAD patients. **B–D** Differences in ICB immunotherapy response, EaSIeR immune score and TIDE score between the low-risk and high-risk groups. **E–H** Influence of immune cells, cell communication, transcription factors and ligand receptor pairs on ICB treatment response

We found that the objective response rate of anti-PD-L1 therapy in the low-risk group was significantly higher than that in the high-risk group (bilateral chi-square test, P=1.295e-10, Fig. 8A, B), and the TIDE score in the high-risk group was significantly higher than that in the low-risk group (Fig. 8D), indicating that patients in the high-risk group were more likely to develop immune evasion, immunotherapy might be less effective, and patients in the low-risk group were more likely to benefit from ICB treatment. The results of the "EaSIeR" package showed that the EaSIeR immune response score of the low-risk group was higher than that of the high-risk group, further indicating that the ICB treatment effect was better in the low-risk group (Fig. 8C). In addition, we found that in terms of immune cell composition, CD8 T cells and Treg T cells were positively correlated with the ICB treatment response (Fig. 8E). In terms of cellular communication, CD4 T-cell-endothelial cells and cancer cell-neutrophils were negatively correlated with the ICB treatment response, while natural killer cells-dendritic cells, neutrophil-B cells and natural killer cells-natural killer cells were positively correlated with the ICB treatment response (Fig. 8F). The transcription factors *STAT4*, *STAT1* and *IRF1* were positively correlated with the ICB response, while *CDX2* and *TP63* were negatively correlated with the ICB response (Fig. 8G). The receptor pairs *CXCL10-SDC4, IFNG-IFNGR1-IFNGR2, CCL5-SDC4 and CXCL16-CXCR6* were positively correlated with the therapeutic response to ICB (Fig. 8H).

### Predicting potential anticancer drugs

**Fig. 9:**
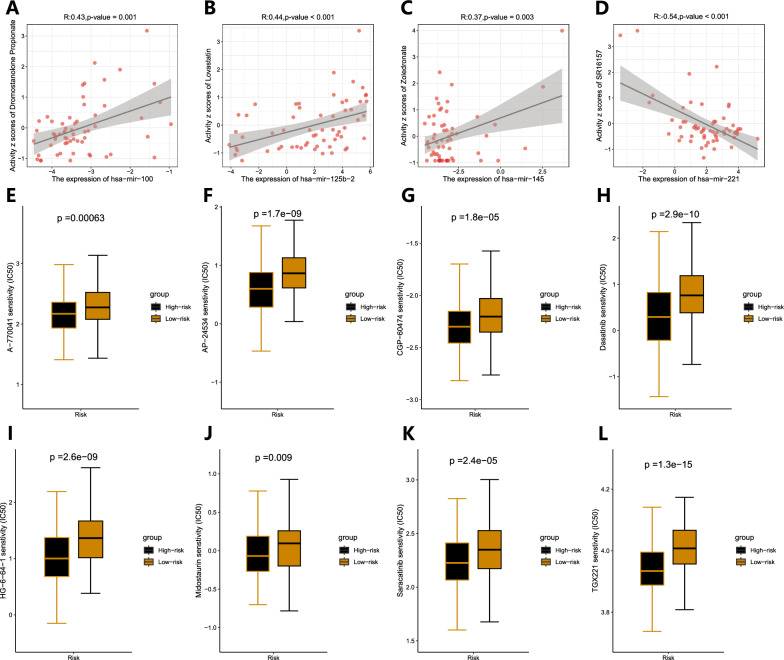
Screening of potential antineoplastic drugs based on prognostic miRNAs and risk group. **A–C** hsa-mir-100, hsa-mir-125b-2 and hsa-mir-145 were positively correlated with sensitivity to dromostanolone propionate, lovastatin and zoledronate, respectively. **D** hsa-mir-221 was negatively correlated with sensitivity to SR16157. **E–L** Eight drugs with higher sensitivity in the high-risk group

To further explore the clinical application of prognostic miRNAs, we used the CellMiner database to explore the relationship between prognostic miRNAs and drug sensitivity (Additional file 4: Table S4). We found that the expression of hsa-mir-100 was positively correlated with sensitivity to dromostanolone propionate (correlation=0.43, P=0.001) (Fig. 9A), the expression of hsa-mir-125b-2 was positively correlated with sensitivity to lovastatin (correlation=0.44, P<0.001) (Fig. 9B), and the expression of hsa-mir-145 was positively correlated with sensitivity to zoledronate (correlation=0.37, P=0.003) (Fig. 9C). The expression of hsa-mir-221 was negatively correlated with sensitivity to SR16157 (correlation=0.54, P<0.001) (Fig. 9D). Because the prognosis of patients in the high-risk group was significantly poorer than that in the low-risk group, we predicted eight drugs with higher sensitivity in the high-risk group: AME 770041, AP-24534, CGP-60474, dasatinib, HG-6-64-1, midostaurin, saracatinib, and TGX221 (Fig. 9E–L).

## Discussion

STAD is the most common histological type of gastric cancer, and despite significant improvements in patient survival in recent years, it continues to have a high recurrence rate and an unsatisfactory prognosis [6]. STAD is mostly asymptomatic until its early stages and lacks effective screening methods for early detection, so many patients are diagnosed at advanced stages [39]. Treatment by endoscopic or limited surgical resection is the most promising treatment for patients with localized STAD [2]. Targeted therapies are more effective than chemotherapy for STAD patients; for example, trastuzumab and apatinib have been approved for the treatment of STAD patients, and the development of more effective drugs and the search for biomarkers with greater sensitivity and specificity remain the main challenges for STAD-targeted therapy [40]. Studies have shown that memory B cells are enriched in the tumours of ICB treatment responders, suggesting that memory B cells are of great importance for the development of biomarkers and targeted therapies [41]. In STAD, miRNAs regulate different signalling pathways, target genes involved in cell migration, angiogenesis and cell proliferation, and play an important role in either cancer promotion or suppression [42–46]. Therefore, it is essential to identify signatures of memory B-cell-associated miRNAs that can be used to predict the prognosis of STAD patients to guide individualized tumour treatment.

In the present study, we constructed a prognostic profile of memory B-cell-associated miRNAs in STAD patients by combining scRNA-seq and bulk RNA-seq analysis. We obtained scRNA-seq data from GSE163558 for three primary gastric cancers and identified memory B-cell-associated genes by a single-cell sequencing data analysis process and reference to the uploader. GO enrichment analysis showed that memory B-cell-associated genes were enriched in cytoplasmic translation, ribosomal protein complex generation, noncoding RNA processing, rRNA metabolism and processing. KEGG enrichment analysis showed that these genes were involved in pathways such as ribosomes, shear bodies, antigen processing and presentation, and IgA production by the intestinal immune network. Memory B-cell-associated genes were enriched in cytoplasmic translation, ribosomal protein complex generation, and noncoding RNA processing. Then, we identified 78 memory B-cell-associated miRNAs by constructing an mRNA‒miRNA coexpression network. Transcription factor enrichment analysis showed that they were mainly enriched in *EP300*, *MAX*, *ESR1*, *ERG*, *BRD4*, *MYC*, *FOXA1*, *HIF1A*, *RUNX1*, *CTCF* and other transcription factors. These transcription factors play an important role in the progression of GC; for example, *EP300* has the ability to regulate the expression of *COL1A2* to control the drug resistance of gastric cancer cells to apatinib [47]. *BRD4* activates *C-MYC* through transcriptional and epigenetic regulation, which increases the proliferation of gastric cancer cells and inhibits the apoptosis of gastric cancer cells [48].

We identified four memory B-cell-associated miRNAs (hsa-mir-221, hsa-mir-100, hsa-mir-145, and hsa-mir-125b-2) significantly associated with prognosis by univariate Cox, random survival forest, and stepwise multiple regression analyses and constructed a prognostic risk model based on these four miRNAs. The overexpression target of hsa-mir-221 in gastric cancer cells has been reported to downregulate the expression of hepatocyte growth factor activator inhibitor type 1 protein, thereby enhancing cell proliferation and migration [49]. Hsa-mir-100, hsa-mir-145 and hsa-mir-125b-2 are tumour suppressors of gastric cancer and regulate potential signalling pathways for gastric cancer cell proliferation, apoptosis and metastasis; for example, hsa-mir-100 antagonism increases the expression level of *HS3ST2*, the target gene of hsa-mir-100, leading to the activation of the Notch apoptotic pathway in tumour cells to suppress the development of gastric cancer [50]. Upregulation of hsa-mir-145 inhibits gastric cancer cell proliferation, increases apoptosis and blocks the cell cycle in G1 phase [51]. The expression of hsa-mir-125b-2 decreases cell viability and colony formation, promotes apoptosis and inhibits the migration and invasion of gastric adenocarcinoma cells, in addition to targeting the downregulation of *PIK3CB* expression [52]. Interestingly, among these miRNAs, hsa-mir-221 was expressed at low levels in the high-risk group, and hsa-mir-100, hsa-mir-145 and hsa-mir-125b-2 were highly expressed in the high-risk group.

The risk scores of patients were calculated according to the prognostic characteristics, and patients were divided into high-risk and low-risk groups based on the median risk score value. We found that the prognosis of patients in the high-risk group was significantly worse than that in the low-risk group. The accuracy and stability of this risk score model were also validated in two internal validation cohorts. Both univariate Cox regression analysis and multivariate Cox regression analysis showed that the RS could predict the prognosis of STAD patients independently of other clinical indicators and had good performance. We further constructed a nomogram containing the risk score and clinicopathological indicators of patients to provide a metric for assessing the prognosis of patients from multiple aspects and used calibration curves to assess the predictive ability of the nomogram. The results showed that our nomogram has a superior predictive ability for OS.

The TME plays a key role in the anticancer response and can significantly affect prognosis [53]. We further explored the relationship between the RS and TME. We found that the immune score, stromal score and ESTIMATE score were significantly higher in the high-risk group than in the low-risk group, which may represent a higher level of immune cell infiltration in the TME of STAD patients. In addition, compared with the low-risk group, patients in the high-risk group had a higher proportion of naïve B cells, monocytes, M2 macrophages, resting dendritic cells, and resting mast cells, whereas activated memory CD4 T cells, follicular helper T cells, resting NK cells, and neutrophils had higher proportions in the low-risk group. M2 macrophages have preneoplastic characteristics and promote tumour growth and metastasis [54]. Dendritic cells are the most potent antigen-presenting cells [55]. An increased percentage of NK cells in tumour tissues may indicate a better prognosis [56]. Thus, the differences in TME revealed by risk grouping based on our model suggest that different prognoses of STAD patients are likely to arise from heterogeneity in the TME.

Cancer immunotherapy, represented by ICB therapy, has become a mainstream treatment modality for malignancies, and TIDE characteristics have the ability to show the effectiveness of ICB therapy [57]. We found that TIDE scores were significantly higher in the high-risk group than in the low-risk group, suggesting that patients in the high-risk group are more likely to experience immune evasion, leading to ICB treatment failure. Different mechanisms in the TME are involved in mediating the immune response and influencing the efficacy of ICB treatment [58]. The EaSIeR results showed that immune response scores were lower in the high-risk group than in the low-risk group, indicating that ICB treatment was less effective in the high-risk group. Consistent with expectations, we further explored the effects of cellular components, cellular communication, transcription factors and ligand receptors in the TME of STAD patients on ICB treatment response and found that CD8 T cells, neutrophil-B cells, NK cells-CD8 T cells, and *STAT4-SDC4* were strong positive biomarkers of ICB treatment response in STAD patients. Consistent with the results of previous studies, CD8 T cells play an important role in the recognition and killing of tumour cells [59]. Neutrophils are able to directly secrete B-cell activating factors to regulate B cells and enhance the immune response [60]. NK cell-induced dendritic cells drive the initiation of type 17 CD8 T cells with the ability to produce IFN-γ and interleukin-17A [61]. *STAT4*-mediated miR-3619-5p controls the onset and progression of STAD by regulating *TBC1D10B* expression, and STAD patients with high *STAT4* expression are predicted to have better clinical outcomes [62, 63]. The interaction of *STAT4* and *SDC4* in the TME may recruit fibroblasts, which in turn inhibit immunotherapeutic responses [64, 65].

Finally, we also predicted potential anticancer drugs. The results showed that dromostanolone propionate, lovastatin, and zoledronate were positively correlated with the expression of hsa-mir-100, hsa-mir-125b-2, and hsa-mir-145, respectively. However, SR16157 was negatively correlated with the expression of hsa-mir-221. In addition, our risk model classified the high-risk group with a poor prognosis, and we screened drugs that STAD was more sensitive to for the high-risk group, with the aim of guiding drug treatment decisions for these patients. The STAD of the high-risk group had the highest sensitivity to CGP-60474 among the eight drugs screened. A previous study showed that CGP-60474 is a potent inhibitor of cell cycle protein-dependent kinases and is associated with the control of cell cycle transition [66].

Although the risk model obtained in this study has a good ability to predict prognosis, it still has some limitations. For example, all the data in this study were from public databases and were retrospective, and the stability of the predictive model must be further confirmed in a prospective cohort study. The results of drug sensitivity need to be further verified by cell experiments.

## Conclusion

In summary, we constructed a novel prognostic signature of memory B-cell-associated miRNAs for STAD based on scRNA-seq and bulk RNA-seq data. This signature reflects the TME, immunotherapy response, prognosis of STAD patients and provides some prognostic insights into individualized and precise treatment for STAD patients. In future research, we can further explore the specific regulatory mechanism and biological functions of the prognostic miRNAs and their target genes in STAD patients. Confirmatory experiments can also be conducted on the predictive ability of the RS model and the therapeutic effect of the drug CGP-60474 in clinical patients.

### Supplementary Information

**Additional file 1: Table S1.** Memory B-cell marker genes.

**Additional file 2: Table S2.** mRNA‒miRNA coexpression network.

**Additional file 3: Table S3.** Clinical characteristics summary descriptive table grouped by the risk score.

**Additional file 4: Table S4.** CellMiner results.

**Additional file 5: Table S5.** Databases and tools used in this article.

## Data Availability

All data generated or analysed during this study are included in the Methods section and Additional file 5: Table S5 (a list of databases and tools).

## Abbreviations

miRNA: MicroRNA
GAC: Gastric adenocarcinoma
STAD: Stomach adenocarcinoma
scRNA-seq: Single cell RNA sequencing
RSF: Random survival forest
StepCox: Stepwise multiple Cox regression
OS: Overall survival
ROC: Time-dependent receiver operating characteristic
K‒M: Kaplan‒Meier
GC: Gastric cancer
TME: Tumour microenvironment
GEO: Gene expression omnibus
TCGA: The cancer genome atlas
PCA: Principal component analysis
UMAP: Uniform manifold approximation and projection
RS: Risk score
TIDE: Tumour immune dysfunction and exclusion
ICB: Immune checkpoint blockade
TMB: Tumour mutational burden
IC50: Semi-maximum inhibitory concentration
GO: Gene ontology
KEGG: Kyoto encyclopedia of genes and genomes
CIs: Confidence intervals
M stage: Tumour metastasis
N stage: Lymph node involvement
T stage: Tumour infiltration
Stage: Tumour grade

## References

[1] Sung H, Ferlay J, Siegel RL, Laversanne M, Soerjomataram I, Jemal A (2021). Global cancer statistics 2020: GLOBOCAN estimates of Incidence and mortality Worldwide for 36 cancers in 185 countries. CA Cancer J Clin.

[2] Ajani JA, Lee J, Sano T, Janjigian YY, Fan D, Song S (2017). Gastric adenocarcinoma. Nat Rev Dis Primers.

[3] Karimi P, Islami F, Anandasabapathy S, Freedman ND, Kamangar F (2014). Gastric cancer: descriptive epidemiology, risk factors, screening, and prevention. Cancer Epidemiol Biomarkers Prev.

[4] Chia NY, Tan P (2016). Molecular classification of gastric cancer. Ann Oncol.

[5] Gastric Cancer Association CA-CA (2021). Chinese expert consensus on perioperative treatment of locally advanced gastric cancer (2021 version). Zhonghua Wei Chang Wai Ke Za Zhi.

[6] Smyth EC, Nilsson M, Grabsch HI, van Grieken NC, Lordick F (2020). Gastric cancer. Lancet.

[7] Rihawi K, Ricci AD, Rizzo A, Brocchi S, Marasco G, Pastore LV (2021). Tumour-associated macrophages and inflammatory microenvironment in gastric cancer: novel translational implications. Int J Mol Sci..

[8] Alsina M, Arrazubi V, Diez M, Tabernero J (2023). Current developments in gastric cancer: from molecular profiling to treatment strategy. Nat Rev Gastroenterol Hepatol.

[9] Ruan K, Fang X, Ouyang G (2009). MicroRNAs: novel regulators in the hallmarks of human cancer. Cancer Lett.

[10] Zhang BG, Li JF, Yu BQ, Zhu ZG, Liu BY, Yan M (2012). microRNA-21 promotes tumour proliferation and invasion in gastric cancer by targeting PTEN. Oncol Rep.

[11] Guo SL, Peng Z, Yang X, Fan KJ, Ye H, Li ZH (2011). miR-148a promoted cell proliferation by targeting p27 in gastric cancer cells. Int J Biol Sci.

[12] Sun M, Liu XH, Li JH, Yang JS, Zhang EB, Yin DD (2012). MiR-196a is upregulated in gastric cancer and promotes cell proliferation by downregulating p27(kip1). Mol Cancer Ther.

[13] He C, Wang L, Zhang J, Xu H (2017). Hypoxia-inducible microRNA-224 promotes the cell growth, migration and invasion by directly targeting RASSF8 in gastric cancer. Mol Cancer.

[14] Tsukamoto Y, Nakada C, Noguchi T, Tanigawa M, Nguyen LT, Uchida T (2010). MicroRNA-375 is downregulated in gastric carcinomas and regulates cell survival by targeting PDK1 and 14-3-3zeta. Cancer Res.

[15] Wu H, Huang M, Cao P, Wang T, Shu Y, Liu P (2012). MiR-135a targets JAK2 and inhibits gastric cancer cell proliferation. Cancer Biol Ther.

[16] Kang W, Tong JH, Lung RW, Dong Y, Zhao J, Liang Q (2015). Targeting of YAP1 by microRNA-15a and microRNA-16-1 exerts tumour suppressor function in gastric adenocarcinoma. Mol Cancer.

[17] Hu Q, Hong Y, Qi P, Lu G, Mai X, Xu S (2021). Atlas of breast cancer infiltrated B-lymphocytes revealed by paired single-cell RNA-sequencing and antigen receptor profiling. Nat Commun.

[18] Wang K, Liu J, Li J (2018). IL-35-producing B cells in gastric cancer patients. Medicine (Baltimore).

[19] Hollern DP, Xu N, Thennavan A, Glodowski C, Garcia-Recio S, Mott KR (2019). B cells and T follicular helper cells mediate response to checkpoint inhibitors in high mutation burden mouse models of breast cancer. Cell.

[20] Griss J, Bauer W, Wagner C, Simon M, Chen M, Grabmeier-Pfistershammer K (2019). B cells sustain inflammation and predict response to immune checkpoint blockade in human melanoma. Nat Commun.

[21] Willsmore ZN, Harris RJ, Crescioli S, Hussein K, Kakkassery H, Thapa D (2020). B cells in patients with melanoma: implications for treatment with checkpoint inhibitor antibodies. Front Immunol.

[22] Budczies J, Kirchner M, Kluck K, Kazdal D, Glade J, Allgauer M (2021). A gene expression signature associated with B cells predicts benefit from immune checkpoint blockade in lung adenocarcinoma. Oncoimmunology.

[23] Papalexi E, Satija R (2018). Single-cell RNA sequencing to explore immune cell heterogeneity. Nat Rev Immunol.

[24] Yang J, Wu Z, Wu X, Chen S, Xia X, Zeng J (2022). Constructing and validating of m6a-related genes prognostic signature for stomach adenocarcinoma and immune infiltration: potential biomarkers for predicting the overall survival. Front Oncol.

[25] Hong X, Zhuang K, Xu N, Wang J, Liu Y, Tang S (2022). An integrated analysis of prognostic mRNA signature in early- and progressive-stage gastric adenocarcinoma. Front Mol Biosci.

[26] Xu P, Liu S, Song S, Yao X, Li X, Zhang J (2022). Identification and validation of a novel angiogenesis-related gene signature for predicting prognosis in gastric adenocarcinoma. Front Oncol.

[27] Jiang H, Yu D, Yang P, Guo R, Kong M, Gao Y (2022). Revealing the transcriptional heterogeneity of organ-specific metastasis in human gastric cancer using single-cell RNA Sequencing. Clin Transl Med.

[28] Wang Z, Jensen MA, Zenklusen JC (2016). A practical guide to the cancer genome atlas (TCGA). Methods Mol Biol.

[29] Stuart T, Butler A, Hoffman P, Hafemeister C, Papalexi E, Mauck WM (2019). Comprehensive integration of single-cell data. Cell.

[30] Ning S, Wu J, Pan Y, Qiao K, Li L, Huang Q (2022). Identification of CD4(+) conventional T cells-related lncRNA signature to improve the prediction of prognosis and immunotherapy response in breast cancer. Front Immunol.

[31] Shannon P, Markiel A, Ozier O, Baliga NS, Wang JT, Ramage D (2003). Cytoscape: a software environment for integrated models of biomolecular interaction networks. Genome Res.

[32] Kern F, Fehlmann T, Solomon J, Schwed L, Grammes N, Backes C (2020). miEAA 2.0: integrating multi-species microRNA enrichment analysis and workflow management systems. Nucleic Acids Res.

[33] Yoshihara K, Shahmoradgoli M, Martinez E, Vegesna R, Kim H, Torres-Garcia W (2013). Inferring tumour purity and stromal and immune cell admixture from expression data. Nat Commun.

[34] Chen B, Khodadoust MS, Liu CL, Newman AM, Alizadeh AA (2018). Profiling tumor infiltrating immune cells with CIBERSORT. Methods Mol Biol.

[35] Fu J, Li K, Zhang W, Wan C, Zhang J, Jiang P (2020). Large-scale public data reuse to model immunotherapy response and resistance. Genome Med.

[36] Lapuente-Santana O, van Genderen M, Hilbers PAJ, Finotello F, Eduati F (2021). Interpretable systems biomarkers predict response to immune-checkpoint inhibitors. Patterns.

[37] Reinhold WC, Sunshine M, Liu H, Varma S, Kohn KW, Morris J (2012). Cell miner: a web-based suite of genomic and pharmacologic tools to explore transcript and drug patterns in the NCI-60 cell line set. Cancer Res.

[38] Geeleher P, Cox N, Huang RS (2014). pRRophetic: an R package for prediction of clinical chemotherapeutic response from tumor gene expression levels. PLoS ONE.

[39] Wagner AD, Unverzagt S, Grothe W, Kleber G, Grothey A, Haerting J (2010). Chemotherapy for advanced gastric cancer. Cochrane Database Syst Rev.

[40] Lei ZN, Teng QX, Tian Q, Chen W, Xie Y, Wu K (2022). Signaling pathways and therapeutic interventions in gastric cancer. Signal Transduct Target Ther.

[41] Helmink BA, Reddy SM, Gao J, Zhang S, Basar R, Thakur R (2020). B cells and tertiary lymphoid structures promote immunotherapy response. Nature.

[42] Bou Kheir T, Futoma-Kazmierczak E, Jacobsen A, Krogh A, Bardram L, Hother C (2011). miR-449 inhibits cell proliferation and is down-regulated in gastric cancer. Mol Cancer.

[43] Tsai MM, Wang CS, Tsai CY, Huang HW, Chi HC, Lin YH (2016). Potential diagnostic, prognostic and therapeutic targets of microRNAs in human gastric cancer. Int J Mol Sci.

[44] Chang L, Guo F, Wang Y, Lv Y, Huo B, Wang L (2014). MicroRNA-200c regulates the sensitivity of chemotherapy of gastric cancer SGC7901/DDP cells by directly targeting RhoE. Pathol Oncol Res.

[45] Kogo R, Mimori K, Tanaka F, Komune S, Mori M (2011). Clinical significance of miR-146a in gastric cancer cases. Clin Cancer Res.

[46] Zheng L, Pu J, Qi T, Qi M, Li D, Xiang X (2013). miRNA-145 targets v-ets erythroblastosis virus E26 oncogene homolog 1 to suppress the invasion, metastasis, and angiogenesis of gastric cancer cells. Mol Cancer Res.

[47] Yu G, Chen W, Li X, Yu L, Xu Y, Ruan Q (2022). TWIST1-EP300 expedites gastric cancer cell resistance to Apatinib by activating the expression of COL1A2. Anal Cell Pathol (Amst).

[48] Ba M, Long H, Yan Z, Wang S, Wu Y, Tu Y (2018). BRD4 promotes gastric cancer progression through the transcriptional and epigenetic regulation of c-MYC. J Cell Biochem.

[49] Ning T, Zhang H, Wang X, Li S, Zhang L, Deng T (2017). miR-221 and miR-222 synergistically regulate hepatocyte growth factor activator inhibitor type 1 to promote cell proliferation and migration in gastric cancer. Tumour Biol.

[50] Yang G, Gong Y, Wang Q, Wang Y, Zhang X (2015). The role of miR-100-mediated Notch pathway in apoptosis of gastric tumor cells. Cell Signal.

[51] Wang J, Sun Z, Yan S, Gao F (2019). Effect of miR-145 on gastric cancer cells. Mol Med Rep.

[52] Riquelme I, Tapia O, Leal P, Sandoval A, Varga MG, Letelier P (2016). miR-101-2, miR-125b-2 and miR-451a act as potential tumor suppressors in gastric cancer through regulation of the PI3K/AKT/mTOR pathway. Cell Oncol (Dordr).

[53] Pitt JM, Marabelle A, Eggermont A, Soria JC, Kroemer G, Zitvogel L (2016). Targeting the tumor microenvironment: removing obstruction to anticancer immune responses and immunotherapy. Ann Oncol.

[54] Murray PJ, Wynn TA (2011). Protective and pathogenic functions of macrophage subsets. Nat Rev Immunol.

[55] Banchereau J, Briere F, Caux C, Davoust J, Lebecque S, Liu YJ (2000). Immunobiology of dendritic cells. Annu Rev Immunol.

[56] Ishigami S, Natsugoe S, Tokuda K, Nakajo A, Xiangming C, Iwashige H (2000). Clinical impact of intratumoral natural killer cell and dendritic cell infiltration in gastric cancer. Cancer Lett.

[57] Jiang P, Gu S, Pan D, Fu J, Sahu A, Hu X (2018). Signatures of T cell dysfunction and exclusion predict cancer immunotherapy response. Nat Med.

[58] Lapuente-Santana O, Eduati F (2020). Toward systems biomarkers of response to immune checkpoint blockers. Front Oncol.

[59] Chen DS, Mellman I (2013). Oncology meets immunology: the cancer-immunity cycle. Immunity.

[60] Parsa R, Lund H, Georgoudaki AM, Zhang XM, Ortlieb Guerreiro-Cacais A, Grommisch D (2016). BAFF-secreting neutrophils drive plasma cell responses during emergency granulopoiesis. J Exp Med.

[61] Clavijo-Salomon MA, Salcedo R, Roy S, Das Neves RX, Dzutsev A, Sales-Campos H (2020). Human NK cells prime inflammatory DC precursors to induce Tc17 differentiation. Blood Adv.

[62] Nishi M, Batsaikhan BE, Yoshikawa K, Higashijima J, Tokunaga T, Takasu C (2017). High STAT4 expression indicates better disease-free survival in patients with gastric cancer. Anticancer Res.

[63] Liu Y, Li J, Wang S, Song H, Yu T (2020). STAT4-mediated down-regulation of miR-3619-5p facilitates stomach adenocarcinoma by modulating TBC1D10B. Cancer Biol Ther.

[64] Jiang D, Liang J, Campanella GS, Guo R, Yu S, Xie T (2010). Inhibition of pulmonary fibrosis in mice by CXCL10 requires glycosaminoglycan binding and syndecan-4. J Clin Invest.

[65] Barrett R, Pure E (2020). Cancer-associated fibroblasts: key determinants of tumor immunity and immunotherapy. Curr Opin Immunol.

[66] Stanetty P, Hattinger G, Schnurch M, Mihovilovic MD (2005). Novel and efficient access to phenylamino-pyrimidine type protein kinase C inhibitors utilizing a Negishi cross-coupling strategy. J Org Chem.

