# Supplementary material for: Identification of memory B-cell-associated miRNA signature to establish a prognostic model in gastric adenocarcinoma
Source: J Transl Med. 2023 Sep 21;21:648. doi: 10.1186/s12967-023-04366-2 (PMC10515266; doi:10.1186/s12967-023-04366-2)
Supplement: Supplementary file 3 — Additional file 3: Table S3. Clinical characteristics summary descriptive table grouped by the risk score. [file 12967_2023_4366_MOESM3_ESM.docx]

**Additional Table S3**

**Clinical characteristics summary descriptive table grouped by the risk score**

| **Clinical characteristics** | **High-risk group** | **Low-risk group** | **p-value** |
| --- | --- | --- | --- |
|  | ***N=178*** | ***N=180*** |  |
| Sex: |  |  | 0.488 |
| female | 60 (33.7%) | 68 (37.8%) |  |
| male | 118 (66.3%) | 112 (62.2%) |  |
| Age: |  |  | 0.358 |
| <65 | 81 (45.5%) | 71 (40.1%) |  |
| >=65 | 97 (54.5%) | 106 (59.9%) |  |
| M: |  |  | 0.380 |
| M0 | 156 (91.8%) | 162 (94.7%) |  |
| M1 | 14 (8.24%) | 9 (5.26%) |  |
| N: |  |  | 0.415 |
| N0 | 49 (28.8%) | 56 (31.6%) |  |
| N1 | 45 (26.5%) | 53 (29.9%) |  |
| N2 | 36 (21.2%) | 39 (22.0%) |  |
| N3 | 40 (23.5%) | 29 (16.4%) |  |
| T: |  |  | 0.295 |
| T1 | 6 (3.43%) | 12 (6.70%) |  |
| T2 | 39 (22.3%) | 39 (21.8%) |  |
| T3 | 76 (43.4%) | 85 (47.5%) |  |
| T4 | 54 (30.9%) | 43 (24.0%) |  |
| Stage: |  |  | ***0.014*** |
| Stage Ⅰ | 16 (9.52%) | 32 (18.3%) |  |
| Stage Ⅱ | 67 (39.9%) | 49 (28.0%) |  |
| Stage Ⅲ | 64 (38.1%) | 79 (45.1%) |  |
| Stage Ⅳ | 21 (12.5%) | 15 (8.57%) |  |
